# Loss of lncRNA MIAT ameliorates proliferation and fibrosis of diabetic nephropathy through reducing E2F3 expression

**DOI:** 10.1111/jcmm.15949

**Published:** 2020-10-03

**Authors:** Ting‐Ting Ji, Ying‐Hui Qi, Xiao‐Ying Li, Bo Tang, Ya‐Kun Wang, Peng‐Xi Zheng, Weiliang Li, Xiaolei Qu, Linhong Feng, Shou‐Jun Bai

**Affiliations:** ^1^ Department of Nephrology Qingpu Branch of Zhongshan Hospital Affiliated to Fudan University Shanghai China; ^2^ Department of Nephrology Shanghai Punan Hospital of Pudong Neww District Shanghai China

**Keywords:** diabetic nephropathy, E2F3, MIAT, miR‐147a

## Abstract

Diabetic nephropathy (DN) is a serious kidney disease resulted from diabetes. Dys‐regulated proliferation and extracellular matrix (ECM) accumulation in mesangial cells contribute to DN progression. In this study, we tested expression level of MIAT in DN patients and mesangial cells treated by high glucose (HG). Up‐regulation of MIAT was observed in DN. Then, functional assays displayed that silence of MIAT by siRNA significantly repressed the proliferation and cycle progression in mesangial cells induced by HG. Meanwhile, we found that collagen IV, fibronectin and TGF‐β1 protein expression was obviously triggered by HG, which could be rescued by loss of MIAT. Then, further assessment indicated that MIAT served as sponge harbouring miR‐147a. Moreover, miR‐147a was decreased in DN, which exhibited an antagonistic effect of MIAT on modulating mesangial cell proliferation and fibrosis. Moreover, bioinformatics analysis displayed that E2F transcription factor 3 (E2F3) could act as direct target of miR‐147a. We demonstrated that E2F3 was greatly increased in DN and the direct binding association between miR‐147a and E2F3 was evidenced using luciferase reporter assay. In summary, our data explored the underlying mechanism of DN pathogenesis validated that MIAT induced mesangial cell proliferation and fibrosis via sponging miR‐147a and regulating E2F3.

## 1 INTRODUCTION

Diabetic nephropathy (DN) is a most serious complications of diabetes, and it is a main cause of death of end‐stage renal disease (ESRD).^1^, ^2^ DN is associated with some specific clinical characteristics, including albuminuria, abnormal glomerular morphology and kidney fibrosis.^3^, ^4^, ^5^ In addition, the variation of glomerulus morphology is a major pathological injury of DN.^6^ The mesangial cells are important component of glomerulus, which can act as the barrier of glomerulus.^7^ In addition, mesangial cells can take charge of matrix proteins synthesis, which are the main cells of the glomerular cells.^8^

LncRNAs have a length of over 200 nucleotides (nts), and they are generated through the transcription of genome.^9^, ^10^ Recently, increasing evidence has proved lncRNAs exhibit vital functions on various human pathogenesis.^11^, ^12^, ^13^ The biological effects of lncRNAs in DN pathogenesis have been identified. For example, MALAT1 can modulate renal tubular epithelial pyroptosis through regulating miR‐23c and ELAVL1.^14^ LINC00968 induces DN pathogenesis via recruiting EZH2 to reduce p21.^15^ Additionally, NR_033515 contributes to DN progression via inducing the proliferation, ECM and EMT processes through targeting miR‐743b‐5p.^16^

MIAT is a highly conserved lncRNA.^17^, ^18^ Recently, it has been found that MIAT can participate in multiple diseases, such as microvascular dysfunction, myocardial infarction and cancers.^18^, ^19^, ^20^ These data indicated MIAT can exhibit possible roles in diabetes. Therefore, we aimed to investigate whether MIAT was a significant regulator in DN.

E2F3, as a famous transcription factor, is a prevalent regulator of various genes. It can exert its functions in the way of transcriptional activation.^21^ It has been reported to be involved in various diseases, including human ovarian cancer, gastric cancer and hepatocellular carcinoma.^22^, ^23^, ^24^ Overexpression of E2F3 can promote proliferation of human β cells without inducing apoptosis.^25^ However, the full action of E2F3 in DN has not been well characterized.

In this current study, we observed that down‐regulation of MIAT reduced mesangial cells proliferation and protein secretion of fibrosis. Mechanically, it was identified that MIAT/miR‐147a/E2F3 axis played a significant role DN progression.

## 2 MATERIALS AND METHODS

### 2.1 Patients

Thirty formalin‐fixed paraffin‐embedded renal biopsy samples were obtained from DN patient of Qingpu Branch of Zhongshan Hospital Affiliated to Fudan University from January 2016 to January 2018. Meanwhile, 30 normal kidney tissues were obtained from normal kidney biopsies to serve as a healthy control group. The Ethics Committee approved the use of tissue samples in our research, and we obtained the written informed consent from each patient.

### 2.2 Cell culture

The human mesangial cells were obtained from the Academy of Life Sciences (Shanghai, China). Cells were incubated in DMEM medium with 2 mM glutamine, 50 mM β‐mercaptoethanol, 20% FBS, penicillin/streptomycin antibiotics. In order to induce LG or HG environment, mesangial cells were stimulated with D‐glucose at 5.5 mmol/L glucose l (low glucose group, LG) or at 25 mmol/L glucose (high glucose group, HG).

### 2.3 Cell transfection

MIAT siRNA and miR‐147a mimics were synthesized by Sangon Biotech1. Over‐expressing MIAT plasmid was constructed by Shanghai R&S Biotechnology Company. Sequence fragment of MIAT was inserted into pcDNA3.1 vector and empty pcDNA3.1 vector was served as control. The mesangial cells were seeded into 24‐well plates and transfected using Lipofectamine 3000 (Invitrogen).

### 2.4 Separation of nuclear and cytoplasmic fractions

Total cellular fractions were divided into cytoplasmic fractions and nuclear fractions using a PARIS kit (Thermo Fisher Scientific, Yokohama) to separate the cytoplasmic and nuclear components before the RNA was isolated.

### 2.5 RNA Fish assay

RNA FISH probe mixture of MIAT, 18S or U6 RNA was labelled using Cy3 from RiboBio. RNA FISH kit was purchased from RiboBio. Nuclei were stained by 6‐diamidino‐2‐phenylindole. The images were observed under a laser scanning confocal microscope.

### 2.6 CCK‐8 proliferation assay

The proliferative capacity was determined by CCK‐8 (Sigma‐Aldrich) assay. Mesangial cells were seeded into 96‐well plates and incubated with 10 mL CCK‐8 agent. A microplate reader (Bio‐Rad) was employed to assess the absorbance of the solution at 590 nm.

### 2.7 EdU proliferation assay

EdU proliferation assay was carried out using Cell‐Light EdU Apollo 567 In Vitro Imaging Kit (Ribobio) based on the manufacturers' recommendation. 100 μL medium with 50 μM EdU was added to the cells for 2 hours and then fixed using 4% paraformaldehyde. Hoechst 33,342 and Apollp reaction cocktail were used. We capture the images using a fluorescence microscopy.

### 2.8 Cycle analysis

Flow cytometry analysis (FACScan; BD Biosciences) was used to do the cycle analysis of mesangial cells. After transfection, cells were collected and fixed using 70% ethanol. Then, the mesangial cells were incubated with propidium iodide at room temperature with no light. Finally, CELL Quest 3.0 software was employed to analyse the cell cycle.

### 2.9 Quantitative real‐time PCR

Total RNA was extracted using TRIzol reagents (Takara Bio). To do mRNA and lncRNA quantification, RNA was reverse transcribed to cDNA using PrimeScript™ RT reagent Kit with gDNA Eraser (Takara Bio). For mature miRNA analyses, reverse transcription was conducted using Mir‐X™ miRNA First Strand Synthesis Kit (Takara Bio). Real‐time PCR reactions were carried out using Takara's SYBR Premix Ex Taq™ II using the LightCycler 480 (Roche). Primer sequences were obtained from GeneCopoeia and provided in Table 1. The 2^−△△^ *^C^* ^t^ method was utilized to do calculation.

**Table 1:** Primers used for real‐time PCR

Genes	Forward (5′‐3′)	Reverse (5′‐3′)
GAPDH	CACCCACTCCTCCACCTTTG	CCACCACCCTGTTGCTGTAG
U6	AGAGAAGATTAGCATGGCCCCTG	ATCCAGTGCAGGGTCCGAGG
MIAT	ATCCTCGAGACAAAGAGCCCTCTGCACTAG	ATCGGATCCGAGCAAATGGAGACAAAGGAC
MALAT1	AAAGCAAGGTCTCCCCACAAG	GGTCTGTGCTAGATCAAAAGGCA
ANRIL	TGCTCTATCCGCCAATCAGG	GGGCCTCAGTGGCACATACC
RIAN	CTGTTGTGCCCTCCCTGGATG	CCAGCTAGGCTGTGTAAATCAT
E2F3	TGACCCAATGGTAGGCACAT	CATCTAGGACCACACCGACA
miR‐147a	CGCGGTGTGTGGAAATGC	AGTGCAGGGTCCGAGGTATT

### 2.10 Immunofluorescence

Cells were fixed using 4% paraformaldehyde in PBS for 10 minutes and permeabilized by 0.1% Triton‐X and 1% BSA in PBS at room temperature. DNA staining was carried out with 4′,6‐diamidino‐2‐phenylindole (DAPI). Fibronectin, collagen IV and TGF‐β1 were detected using the corresponding antibodies (1:1000; Cell Signaling Technology, CST) at 37°C for approximately 16 hours. Imaging was conducted using an ECLIPSE Ni microscope (Nikon).

### 2.11 Western blot

Cells were lysed using cell lysis buffer (Beyotime). Protein samples were then separated on 10% SDS‐PAGE gels and then transferred to nitrocellulose filter membranes. Then, the membranes were incubated with primary antibodies at 4°C for a whole night, including Fibronectin, collagen IV and TGF‐β1 and GAPDH (1:1000; Cell Signaling Technology, CST). Then, an HRP‐conjugated anti‐mouse or anti‐rabbit secondary antibody was followed. Finally, we detected the bands using the ECL substrate (Millipore).

### 2.12 Luciferase reporter assay

Cells (5 × 10^4^ cells in each well) were seeded in 24‐well plates for a whole night. Cells were co‐transfected with pGL3‐MIAT‐WT, pGL3‐MIAT‐MUT, pGL3‐E2F3‐WT or pGL3‐E2F3‐WT reporter plasmids and mimics NC, miR‐147a mimics. After transfection, cells were lysed using passive lysis buffer (Promega) and the luciferase activity was examined using the Dual‐Luciferase Reporter Assay System (Promega).

### 2.13 RNA immunoprecipitation assay

RNA immunoprecipitation (RIP) assay was carried out using a Magna RIP RNA‐Binding Protein Immunoprecipitation kit (Millipore) based on the manufacturer's instructions. In brief, 2 × 10^7^ mesangial cell lysates were treated with magnetic beads conjugated with IgG or human anti‐Ago2 antibody (Millipore). Then, the immunoprecipitated RNA was extracted and tested using qRT‐PCR to confirm the enrichment of binding targets.

### 2.14 Statistical analysis

Statistical analysis was carried out using GraphPad Prism 6 software and SPSS 22.0 software. Differences between two groups were evaluated using Student's *t* test. one‐way analysis of variance was adopted for multiple comparisons. A *P*‐value less than .05 indicated the statistically significance.

## 3 RESULTS

### 3.1 Expression of MIAT was elevated in DN

**Figure 1:**
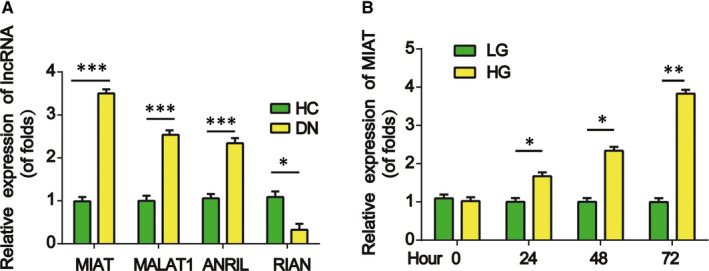
Expression levels of MIAT in DN. A, The expression levels of MIAT were determined using qRT‐PCR in the kidney tissues of DN patients and healthy controls. N = 30 in each group. B, The expression of MIAT was tested in mesangial cells incubated with HG and NG for various hours. Three independent experiments were conducted. Error bars stand for the mean ± SD of at least triplicate experiments. *P < .05, **P < .01, ***P < .001

Firstly, we tested the expression of MIAT, MALAT1, ANRIL and RIAN in DN patient samples. In Figure 1A, MIAT expression was highly up‐regulated in the kidney tissues of DN patients. In addition, as displayed in Figure 1B, MIAT expression was increased in mesangial cells exposed to HG in a time‐dependent manner. These data implied a correlation between MIAT and DN development.

### 3.2 MIAT promoted mesangial cell proliferation

**Figure 2:**
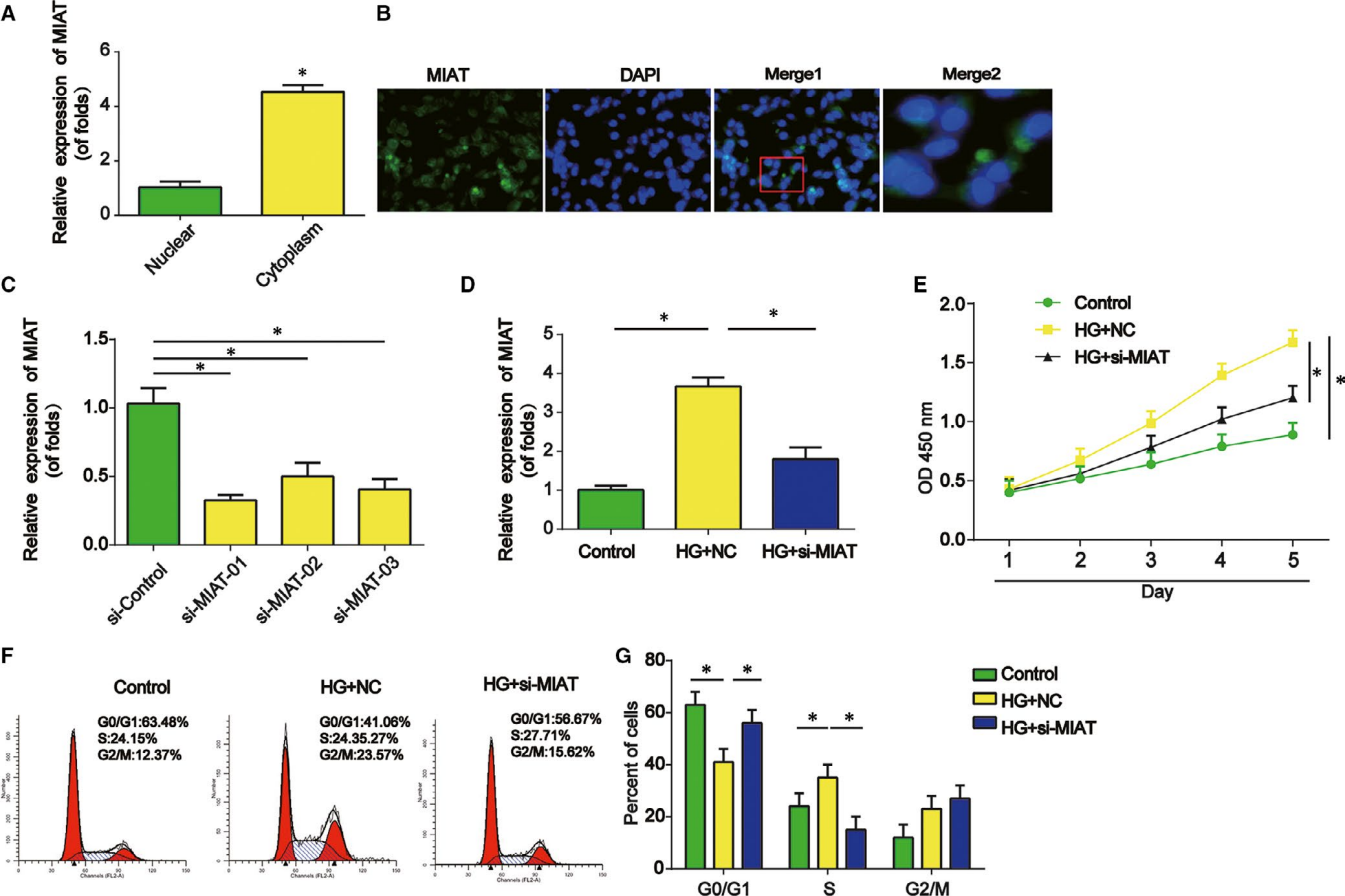
Effect of MIAT on mesangial cell proliferation and cell cycle. A, Expression of MIAT in nucleus and cytoplasm of mesangial cells. B, Expression of MIAT in mesangial cells indicated by FISH assay. C, The expression of MIAT in mesangial cells transfected with MIAT siRNA. D, Expression of MIAT in mesangial cells transfected with MIAT siRNA after HG incubation. E, CCK8 assay was used to test cell survival. F and G, Flow cytometry assay was carried out to test cell cycle. Three independent experiments were conducted. Error bars stand for the mean ± SD of at least triplicate experiments. *P < .05

Then, MIAT levels in the cytoplasm were higher than the levels in the nucleus of mesangial cells as shown in Figure 2A. RNA FISH experiments confirmed the localization of MIAT in mesangial cells. It was indicated that MIAT was highly localized in cytoplasm rather than the nucleus (Figure 2B). We knocked down MIAT expression by transfecting external synthesized siRNA targeting MIAT in mesangial cells (Figure 2C). As shown, siRNA‐01 exhibited a best knock‐down effect. In Figure 2D, MIAT siRNA was able to repress the up‐regulated of MIAT due to high glucose. Cell survival was analysed using CCK‐8. A down‐regulated ratio of cell survival was observed in MIAT silencing mesangial cells, suggesting that MIAT might induce the cell proliferation (Figure 2E). Cycle analysis by flow cytometry indicated that HG accelerated the cycle progression than the NG induced mesangial cells. For another, the si‐MIAT transfection could repress cycle progression (Figure 2F,G).

### 3.3 MIAT enhanced HG‐triggered mesangial cell ECM accumulation

**Figure 3:**
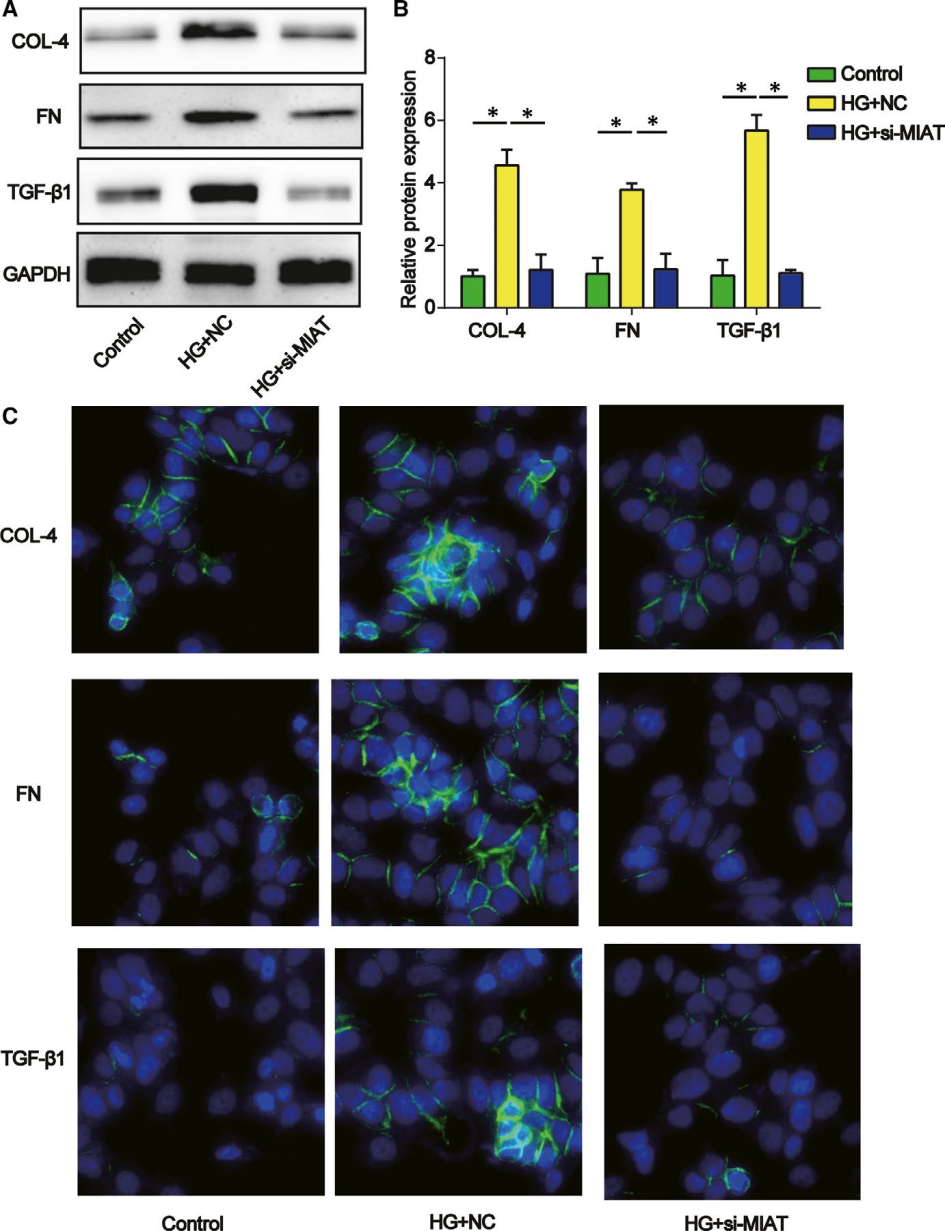
Effect of MIAT on mesangial cell ECM accumulation. A and B, Collagen IV, FN and TGF‐β1 protein expression in mesangial cells transfected with MIAT siRNA. Three independent experiments were conducted. C, Immunofluorescence indicating the expression of collagen IV, FN and TGF‐β1. Error bars stand for the mean ± SD of at least triplicate experiments. *P < .05

Afterwards, Western blot assays were carried out and we illustrated that MIAT silencing by the si‐MIAT transfection dramatically suppressed the ECM proteins (Fibronectin, collagen IV and TGF‐β1) in the HG‐treated mesangial cells (Figure 3A,B). As displayed in Figure 3C, immunofluorescence using fluorescence microscopy indicated that collagen IV, FN and TGF‐β1 were induced by HG, which was reversed by loss of MIAT in mesangial cells.

### 3.4 The target gene of lncRNA MIAT was miR‐147a

**Figure 4:**
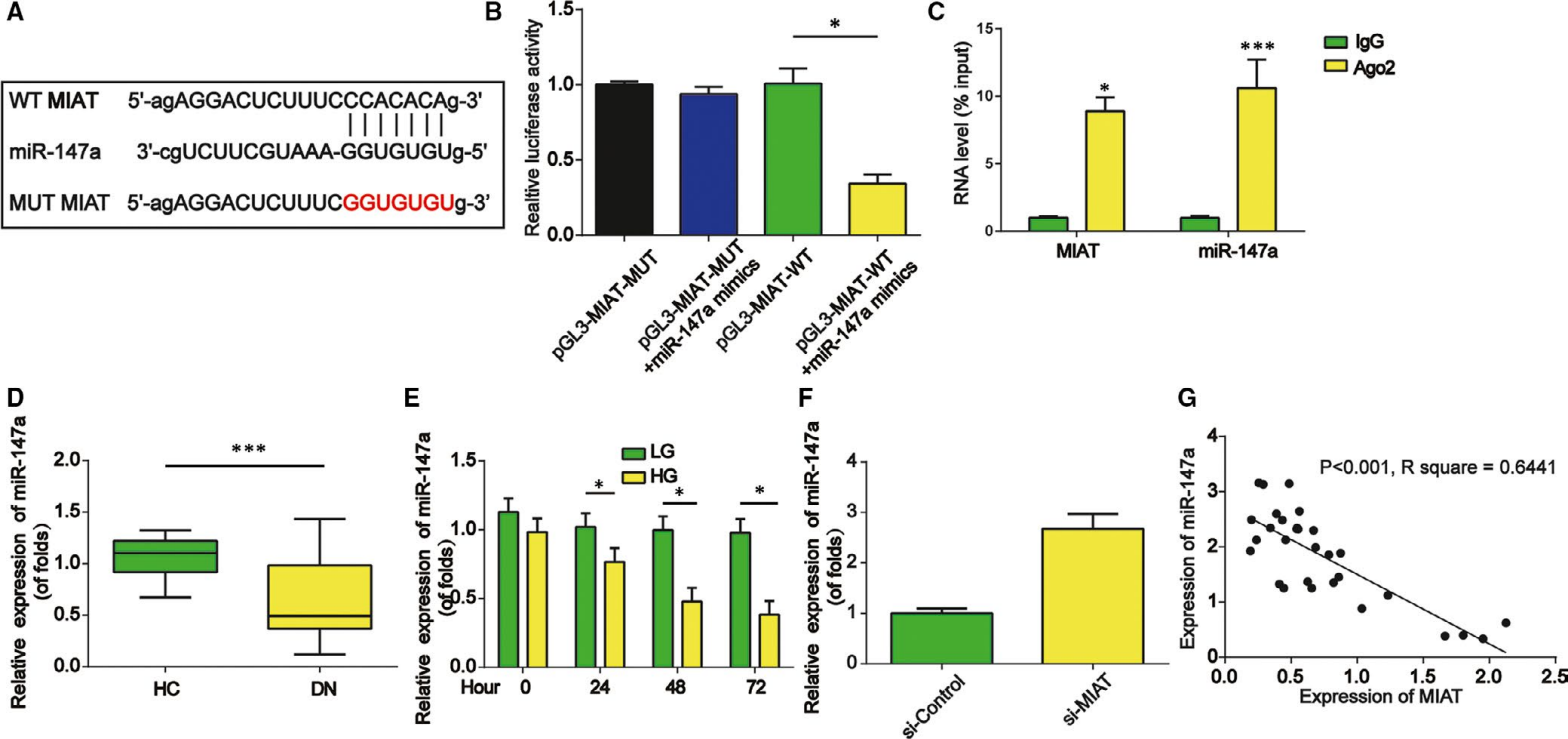
miR‐147a was a direct target of MIAT. A, The luciferase reporter constructs containing the WT‐MIAT or MUT‐MIAT sequence. B, Luciferase activity was tested after co‐transfection of construct containing WT or MUT version of MIAT and miR‐147a mimics. C, Anti‐Ago2 RIP was employed to pull down endogenous RNAs associated with Ago2. IgG was used as the control. The levels of MIAT and miR‐147a were measured by qRT‐PCR. Data were exhibited as fold enrichment in Ago2 relative to input. D, The expression levels of miR‐147a determined using qRT‐PCR in the kidney tissues of DN patients and healthy controls. E, The expression of miR‐147a was tested in mesangial cells incubated with HG and LG for various hours. F, The expression levels of miR‐147a in mesangial cells transfected with MIAT siRNA. G, Correlation analysis of MIAT and miR‐147a expressions in 30 cases of DN patient kidney tissues. Three independent experiments were conducted. Error bars stand for the mean ± SD of at least triplicate experiments. *P < .05, **P < .01, ***P < .001

Then, miR‐147a was predicted as a target of MIAT using bioinformatics algorithm (http://starbase.sysu.edu.cn/). The binding sites between them were confirmed using luciferase reporter assay. Firstly, the luciferase reporter constructs containing the WT‐MIAT or MUT‐MIAT sequence were shown in Figure 4A. Then, mimics of miR‐147a were co‐transfected with construct containing with WT or MUT 3′‐UTR of MIAT. As exhibited in Figure 4B, miR‐147a mimics significantly repressed the luciferase activity of WT‐MIAT but not MUT‐MIAT. As shown in Figure 4C, both MIAT and miR‐147a were specifically enriched in Ago2 antibody‐associated complex, suggesting that miR‐147a is a MIAT‐targeting microRNA. Furthermore, in Figure 4D, miR‐147a expression was repressed in the kidney tissues of DN patients. In Figure 4E, miR‐147a expression was reduced in mesangial cells exposed to HG in a time‐dependent course. For another, we found that loss of MIAT repressed miR‐147a expression in mesangial cells as exhibited in Figure 4F. A negative correlation between miR‐147a and MIAT was observed in DN patients in Figure 4G.

### 3.5 The target gene of miR‐147a was E2F3

**Figure 5:**
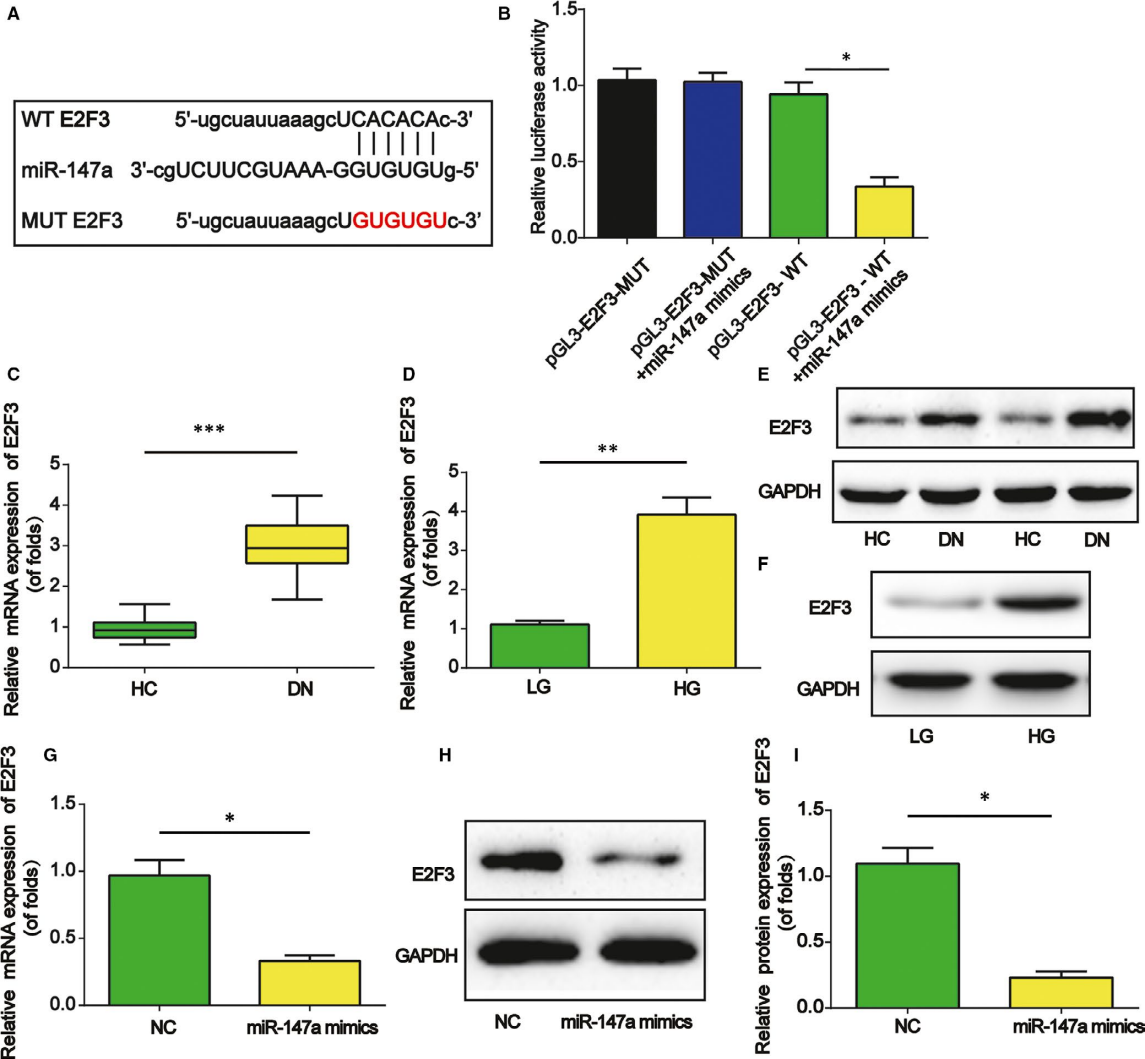
E2F3 was a target of miR‐147a. A, The luciferase reporter constructs containing the WT‐E2F3 or MUT‐E2F3 sequence. B, WT‐E2F3 or MUT‐E2F3 was co‐transfected into mesangial cells with miR‐147a mimics or their corresponding negative controls. C and D, E2F3 mRNA expression in DN. E and F, E2F3 protein expression in DN. G, E2F3 mRNA expression in mesangial cells transfected with miR‐147a mimics. H and I, E2F3 protein expression in mesangial cells. Three independent experiments were conducted. Error bars stand for the mean ± SD of at least triplicate experiments. *P < .05, **P < .01, ***P < .001

Subsequently, E2F3 was predicted as a target of miR‐147a using bioinformatics analysis (http://starbase.sysu.edu.cn/). The luciferase reporter constructs carrying the WT‐E2F3 or MUT‐E2F3 sequence were shown in Figure 5A. Mimics of miR‐147a were co‐transfected with construct containing with WT or MUT 3′‐UTR of E2F3. In Figure 5B, miR‐147a mimics remarkably restrained the luciferase activity of WT‐MIAT but not MUT‐MIAT. Furthermore, in Figure 5C,D, E2F3 mRNA expression was up‐regulated in DN. Consistently, In Figure 5E,F, E2F3 protein expression was also increased in DN. In Figure 5G‐I, E2F3 expression was targeted by miR‐147a mimics in mesangial cells.

### 3.6 miR‐147a reversed the function of MIAT on mesangial cell proliferation and fibrosis

**Figure 6:**
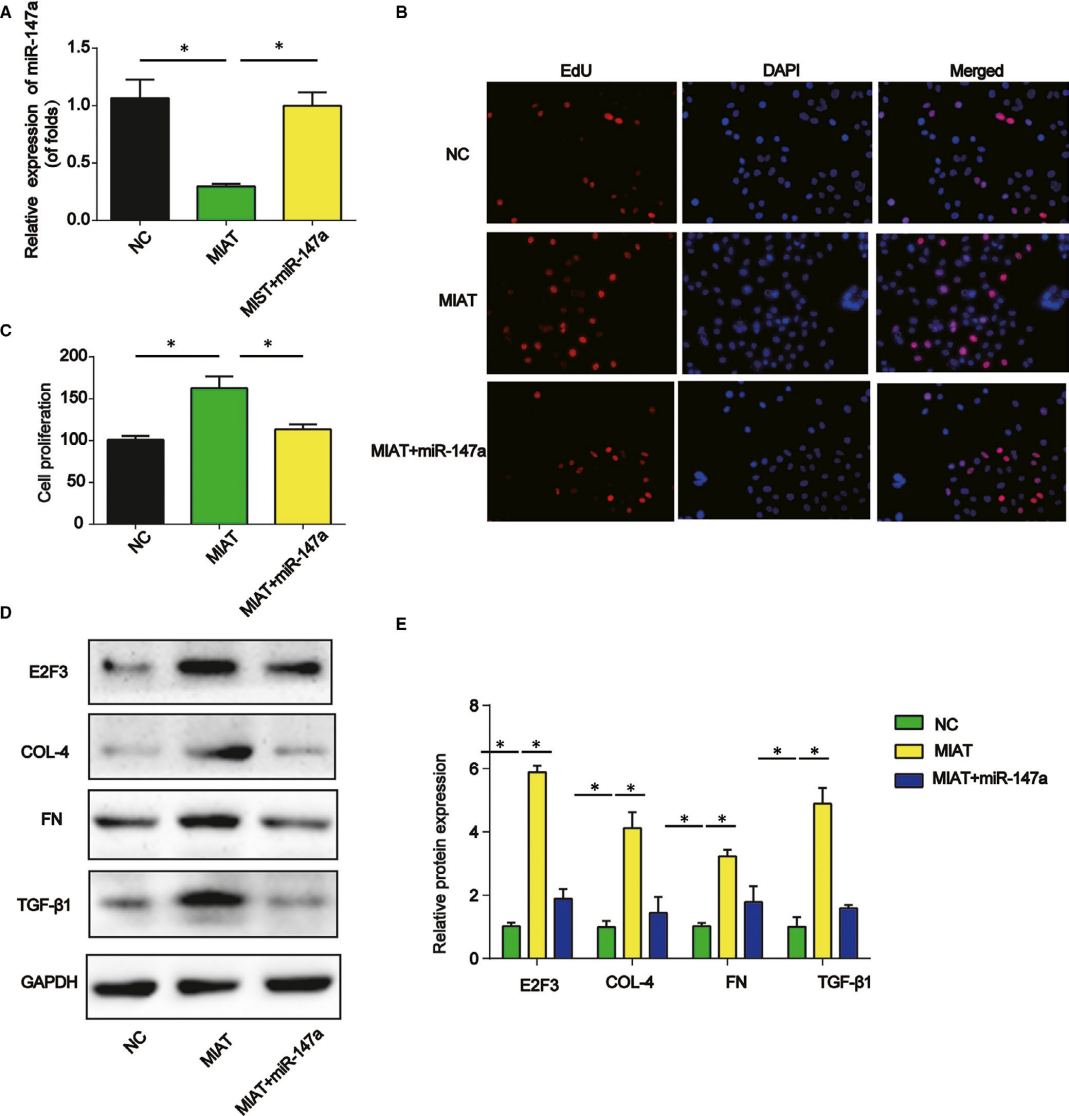
Effect of miR‐147a on mesangial cell proliferation and ECM accumulation. A, The expression of miR‐147a in mesangial cells transfected with MIAT overexpression plasmid and then transfected with miR‐147a mimics. B and C, EdU assay was used to test cell proliferation (D and E) E2F3, collagen IV, FN and TGF‐β1 protein expression in mesangial cells. Three independent experiments were conducted. Error bars stand for the mean ± SD of at least triplicate experiments. *P < .05

Furthermore, after mesangial cells were transfected with MIAT overexpression plasmid, miR‐147a mimics were used. As shown in Figure 6A, miR‐147a was successfully reduced by MIAT overexpression plasmid while increased by miR‐147a mimics. In Figure 6B,C, cell proliferation was analysed using EdU incorporation assay. MIAT greatly increased mesangial cell proliferation, which was reversed by miR‐147a. Then, E2F3, Fibronectin, collagen IV and TGF‐β1 protein expression was also obviously down‐regulated by miR‐147a mimics (Figure 6D,E).

## 4 DISCUSSION

Recently, the increasing evidence has illustrated the important functions of lncRNAs on human diseases.^26^ DN is a lethiferous end‐stage complication of diabetes.^27^, ^28^ LncRNAs can effectively participate in the pathophysiological process of DN.^29^, ^30^ For instance, we found that lncRNA 150Rik can promote proliferation of mesangial cells via regulating miR‐451 and p38 MAPK pathway in DN.^31^ LncRNA MEG3 enhances fibrosis and inflammatory response through modulating miR‐181a, Egr‐1 and TLR4 axis in DN.^32^

In this present research, we concentrated on the mechanism of MIAT in DN. We proved that MIAT was significantly elevated in DN patients, and mesangial cells incubated with HG. Then, our in vitro assays indicated that MIAT involved in DN by inducing cell proliferation and ECM accumulation. Using bioinformatics tools, miR‐147a was identified as the direct binding target of MIAT, and this was validated using luciferase reporter assay. Furthermore, E2F3 could act as a target for miR‐147a and miR‐147a greatly repressed E2F3 expression level in vitro. Next, overexpression of miR‐147a significantly reversed the effect of MIAT on DN progression.

LncRNA MIAT is closely associated with myocardial infarction. A growing number of evidence indicates that MIAT participates in many cellular processes, such as microvascular dysfunction, neurogenic commitment and age‐related cataract.^33^ For example, MIAT serves as a ceRNA to increase DAPK2 through sponging miR‐22‐3p in diabetic cardiomyopathy.^34^ MIAT can act as a biomarker by sponging miR‐29b in diabetic retinopathy.^35^ In addition, MIAT is involved in diabetic optic nerve injury through modulating HSPA5 via binding with miR‐379.^36^ In our study, we found that MIAT was able to target miR‐147a to induce mesangial cell proliferation and ECM contents.

Recently, ceRNA theory has obtained much recognition.^37^ In this theory, lncRNAs function via competing with shared microRNAs, which can play important roles in post‐transcriptional regulation.^38^ It has demonstrated that miR‐147a is involved in some types of cancer.^39^, ^40^ Nevertheless, little is known about the role of miR‐147a in DN. miR‐147a was predicted as a target of MIAT. We found miR‐147a was reduced in DN patients and HG‐treated mesangial cells. A negative correlation between MIAT and miR‐147a was confirmed in the kidney tissues of DN patients. In addition, overexpression of miR‐147a rescued the effect of MIAT on DN progression. In order to fully investigate the molecular mechanism miR‐147a, microRNA inhibitors would be used to strengthen our data. Many other miRNAs can be predicted by using http://starbase.sysu.edu.cn/. Some of them have positive or negative effects on mesangial cell proliferation. In our future study, we would like to do more research on them.

*E2F3* gene is on chromosome 6p22 and has a length of 91.5 kb.^41^ Meanwhile, E2F3 is a major member of E2F family and it can play a crucial role in modulating cell cycle, proliferation and apoptosis. For example, miR‐194‐5p enhances bladder cancer development through targeting E2F3.^42^ In addition, miR‐148a can repress lung adenocarcinoma progression through directly targeting E2F3.^43^ Increase of E2F3 can promote functional human β cells proliferation with no induction of apoptosis.^25^ A previous study reports that miR‐503 increases podocyte injury through targeting E2F3 in DN.^44^ E2F3 was shown to be a target gene for miR‐147a in this work. In this study, we observed the expression of E2F3 was significantly induced in DN. Mimics of miR‐147a significantly repressed E2F3 expression. Hence, the competitive binding of MIAT to miR‐147a release E2F3, which could lead to an enhancement of cell proliferation and fibrosis during DN development. In addition, many other mRNA transcripts can be predicted as the targets for miR‐147a. In our future study, more researches are warranted to work on them.

In conclusion, we reported a novel of MIAT/miR‐147a/E2F3 axis and explored its involvements in DN. Our study identified novel insights for the understandings of the significance of lncRNAs and microRNAs on DN. This facilitated the development of effective therapeutic solutions for DN.

## CONFLICT OF INTEREST

The authors declare that there are no conflicts of interest.

## AUTHOR CONTRIBUTIONS

**Ting‐Ting Ji:** Conceptualization (equal); Methodology (equal); Writing‐original draft (equal). **Ying‐Hui Qi:** Data curation (equal); Software (equal). **Xiao‐Ying Li:** Data curation (equal); Investigation (equal). **Bo Tang:** Investigation (equal); Visualization (equal); Writing‐original draft (equal). **Ya‐kun Wang:** Software (equal); Supervision (equal); Validation (equal). **Peng‐Xi Zheng:** Investigation (equal); Software (equal); Visualization (equal). **Weiliang Li:** Formal analysis (equal); Investigation (equal); Validation (equal). **Xiaolei Qu:** Formal analysis (equal); Supervision (equal); Validation (equal). **Linhong Feng:** Investigation (equal); Methodology (equal); Software (equal). **Shou‐Jun Bai:** Conceptualization (equal); Funding acquisition (equal); Resources (equal); Writing‐review & editing (equal).

## Data Availability

All data in this study were available if requested.
